# A prospective cohort study protocol: monitoring and surveillance of adverse events following heterologous booster doses of Oxford AstraZeneca COVID-19 vaccine in previous recipients of two doses of Sinopharm or Sputnik V vaccines in Iran

**DOI:** 10.1186/s12889-023-16265-8

**Published:** 2023-07-24

**Authors:** Shahin Soltani, Behzad Karami Matin, Mohammad Mehdi Gouya, Sayed Mohsen Zahraei, Ghobad Moradi, Omid Chehri, Moslem Soofi, Mehdi Moradinazar, Fatemeh Khosravi Shadmani, Mahsa Kalantari, Hamidreza Khajeha, Mohammad Hassan Emamian, Farid Najafi

**Affiliations:** 1 Research Center for Environmental Determinants of Health (RCEDH), Health Institute, Kermanshah University of Medical Sciences, Kermanshah, Iran; 2 Social Development and Health Promotion Research Center, Health Institute, Kermanshah University of Medical Sciences, Kermanshah, Iran; 3 Iranian Center for Communicable Diseases Control, Ministry of Health & Medical Education, Tehran, Iran; 4 Nosocomial Infection Research Center, Isfahan University of Medical Sciences, Isfahan, Iran; 5 Social Determinant of the Health Research Center, Research Institute for Health Development, Kurdistan University of Medical Sciences, Sanandaj, Iran; 6 Behavioral Disease Research Center, Kermanshah University of Medical Sciences, Kermanshah, Iran; 7 Ophthalmic Epidemiology Research Center, Shahroud University of Medical Sciences, Shahroud, Iran

**Keywords:** COVID-19, Cohort study, Sinopharm vaccine, AstraZeneca vaccine, Vaccine safety surveillance

## Abstract

**Background:**

Regarding the paucity of evidence on the side effects of the booster dose of Oxford AstraZeneca vaccine in vaccinated people with Sinopharm or Sputnik V, we aimed to set up a cohort event monitoring (CEM) study to capture adverse events occurring in individuals who will receive the booster doses of AstraZeneca (either the first or second booster dose) following being vaccinated with Sinopharm or sputnik V vaccines in Iran.

**Methods:**

The present study is an active COVID-19 vaccine safety surveillance through an observational prospective cohort study that will be conducted in vaccination centers in Iran. The study will be conducted in twelve provinces of Iran. Study sites are vaccination centers where the AstraZeneca vaccine is administered to the cohort population. The study population includes all individuals who have received two doses of Sinopharm or Sputnik V vaccines and either the first or second booster dose of AstraZeneca according to the national guidelines for immunization in Iran in 2023. We are planning to include 30,000 eligible people in this study. Each individual will be followed up for 13 weeks after either the first or second booster dose of the AstraZeneca vaccine. Furthermore, convenience sampling is used to include participants in the present study. Participation in the study will be strictly voluntary.

**Discussion:**

With the planned study we will provide a valid epidemiological evidence to improve the understanding of the safety of the booster dose of the AstraZeneca and to better evaluate the effectiveness of public health interventions. This could help policy makers in managing the COVID-19 pandemic according to scientific evidence.

**Supplementary Information:**

The online version contains supplementary material available at 10.1186/s12889-023-16265-8.

## Background

In December 2019, an outbreak of respiratory disease caused by a novel coronavirus strain was reported in Wuhan City, Hubei Province, China. The novel coronavirus was named ‘severe acute respiratory syndrome coronavirus 2’ (SARS-CoV-2), while the disease associated with it is referred to as COVID-19. The virus quickly spread to different parts of China and other countries in the world [1].

The development of safe and effective vaccines is a vital approach to control of the SARS-CoV-2 pandemic. Providing equitable access to vaccines across the world is one of the critical approaches to mitigating the public health and economic impact of the pandemic [2]. Regarding the literature, vaccine candidates against COVID-19 comprise traditional virus- and protein-based vaccines and newer platforms like mRNA vaccines, viral vector-based- and nucleic acid vaccines [3]. Vaccines confirmed for use in national immunization programs (NIPs), are considered safe and effective based on verifiable evidence from randomized controlled clinical trials. However, studies indicate that vaccines are not entirely free of risks, despite rigorous safety evaluation during clinical development, and occasional adverse events will take place after vaccination at the population level [4–6]. Regarding that vaccines are often suggested for otherwise healthy individuals, the key to the success of NIPs will be public trust in vaccine safety [7].

Therefore, systematic vaccine safety surveillance is necessary for approving the safety of vaccines and public trust, across the world. When plans for immunization with COVID-19 vaccines are set up, there should be a pharmacovigilance systems simultaneously, and implement specific COVID-19 vaccine safety surveillance. In fact, WHO developed such protocol for COVID-19 vaccine safety surveillance manual [8]. Given that routine passive reporting systems might not be adequate to allow quick assessment and appropriate public health response during COVID-19 vaccine introduction; active safety surveillance is recommended.

In Iran, public vaccination with COVID-19 vaccines as part of the NIP was started in 2021. In addition to vaccines such as Sinopharm, Oxford/AstraZeneca ChAdOx1 nCoV-19 adenoviral AstraZeneca, and Sputnik V, Iranian received different types of domestic vaccines. Accruing to the Ministry of Health and Medical Education’s (MOHME) report on COVID-19 on August 16, 2022, the total number of cases, deaths, and recovered cases was 7,488,493; 143,093; and 7,190,330 respectively [9]. Also, 27,685,680 people have received the third dose of the COVID-19 vaccine, while the corresponding value for the second dose was 57,959,218 people [10].

Since the pandemic of COVID-19 and development of vaccines, permitting entry for travelers who have been completely vaccinated has become a common standard for countries across the world to prevent widespread transmission of the coronavirus. However, vaccination against the coronavirus disease and being vaccinated with a vaccine that a destination country (or WHO) accepts it as valid proof of immunity have turned out to be two separate issues. Accordingly, some countries may permit entry only to people who have been vaccinated with a number of COVID-19 vaccines such as the Pfizer-BioNTech, AstraZeneca, Moderna, and Johnson and Johnson vaccines which might be not available for all countries. In Iran, more than 130 million doses of COVID-19 vaccines have been used among the general population. However, the majority of such people received a vaccine that still has not been recognized by international institutions. In addition, some Iranian received vaccines that even have not been considered under EUL by WHO. This group encounters a problem to travel to countries that require a valid vaccine certificate.

In addition, with no full coverage of world population with vaccination against COVID-19, the pandemic is still far from extinction. The arrival of new variants that are most likely to be more transmissible and vaccine evasive as well as the pattern of waning of antibodies, the booster dosing of vaccine is highly recommended by WHO. However, such booster dose of vaccines, whether it is homologous or heterologous might be accompanied with adverse events.

Regarding the paucity of evidence on the adverse event of the booster dose of AstraZeneca vaccine in vaccinated people with Sinopharm and Sputnik V, we aim to set up a cohort event monitoring (CEM) study to capture adverse events occurring in individuals who will receive the booster vaccination with AstraZeneca (either the first or the second booster dose) following being vaccinated with Sinopharm or Sputnik V vaccines in Iran.

## Methods/design

### Objectives

#### General objective

Measuring safety of the booster doses of AstraZeneca vaccine in people vaccinated with two doses of Sinopharm or Sputnik V vaccines in Iran.

#### Specific objectives

1. To Estimate the incidence of different types of serious adverse events (SAEs) within 13 weeks in all enrolled vaccinated individuals after either the first or second booster vaccination with AstraZeneca.
2. To estimate the incidence of different types of adverse events of special Interests (AESIs) within 13 weeks.
3. To estimate the incidence of COVID-19 after either the first or second booster dose of A in all enrolled people.

### Design

The present study is an active COVID-19 vaccine safety surveillance through an observational prospective cohort study that will be conducted in vaccination centers in Iran. The present protocol has been developed based on the COVID-19 vaccine safety guidance manual introduced by the WHO in 2020 [11].

### Study settings

The study will be conducted in all major cities of Iran. Study sites are vaccination centers where the AstraZeneca is administered to the cohort population. Although vaccination against COVID-19 will be done in all health care centers plus other temporary locations, AstraZeneca has been provided in some predefined centers. In each site studied except Tehran, there will be a vaccination center to collect data on people get vaccinated by Astrazeneca. In Tehran, three vaccination centers have been determined to collect such data. Totally, we will collect data in all major cities. Study sites and vaccination centers will be selected by the Ministry of Health and Medical Education (MOHME). In order to receive the previous history of vaccination, we get access to Integrated Public Health System (IPHS). Table 1 shows characteristics of the cohort sites in the present study.

**Table 1:** The main variables of the study gathered in enrolment and follow up questionnaires

Province	Site	Affiliation
Alborz	Alborz	Alborz University of Medical Sciences
Tehran	Tehran (TUMS)	Tehran University of Medical Sciences
Tehran	Tehran (SUMS)	Shahid Beheshti University of Medical Sciences
Tehran	Tehran (IUMS)	Iran University of Medical Sciences
Kermanshah	Kermanshah	Kermanshah University of Medical Sciences
Kurdistan	Sanandaj	Kurdistan University of Medical Sciences
Sistan and Baluchestan	Zahedan	Zahedan University of Medical Sciences
South Khorasan	Birjand	Birjand University of Medical Sciences
Kerman	Kerman	Kerman University of Medical Sciences
Razavi Khorasan	Mashhad	Mashhad University of Medical Sciences
Isfahan	Isfahan	Isfahan University of Medical Sciences
East Azerbaijan	Tabriz	Tabriz University of Medical Sciences
Khuzestan	Ahvaz	Ahvaz Jondishapur University of medical Sciences
Boshehr	Boshehr	Boshehr University of Medical Sciences

### Study period

The time point for enrolment will be either the first or second booster vaccination with AstraZeneca in one of the study sites, after primary vaccination with Sinopharm or Sputnik V.

Each individual will be followed-up for 13 weeks after either the first or second booster vaccination with AstraZeneca vaccine. Thirteen weeks follow-up period was chosen because this covers the most common risk windows for AESIs (42 days).

### Participants

**Fig. 1:**
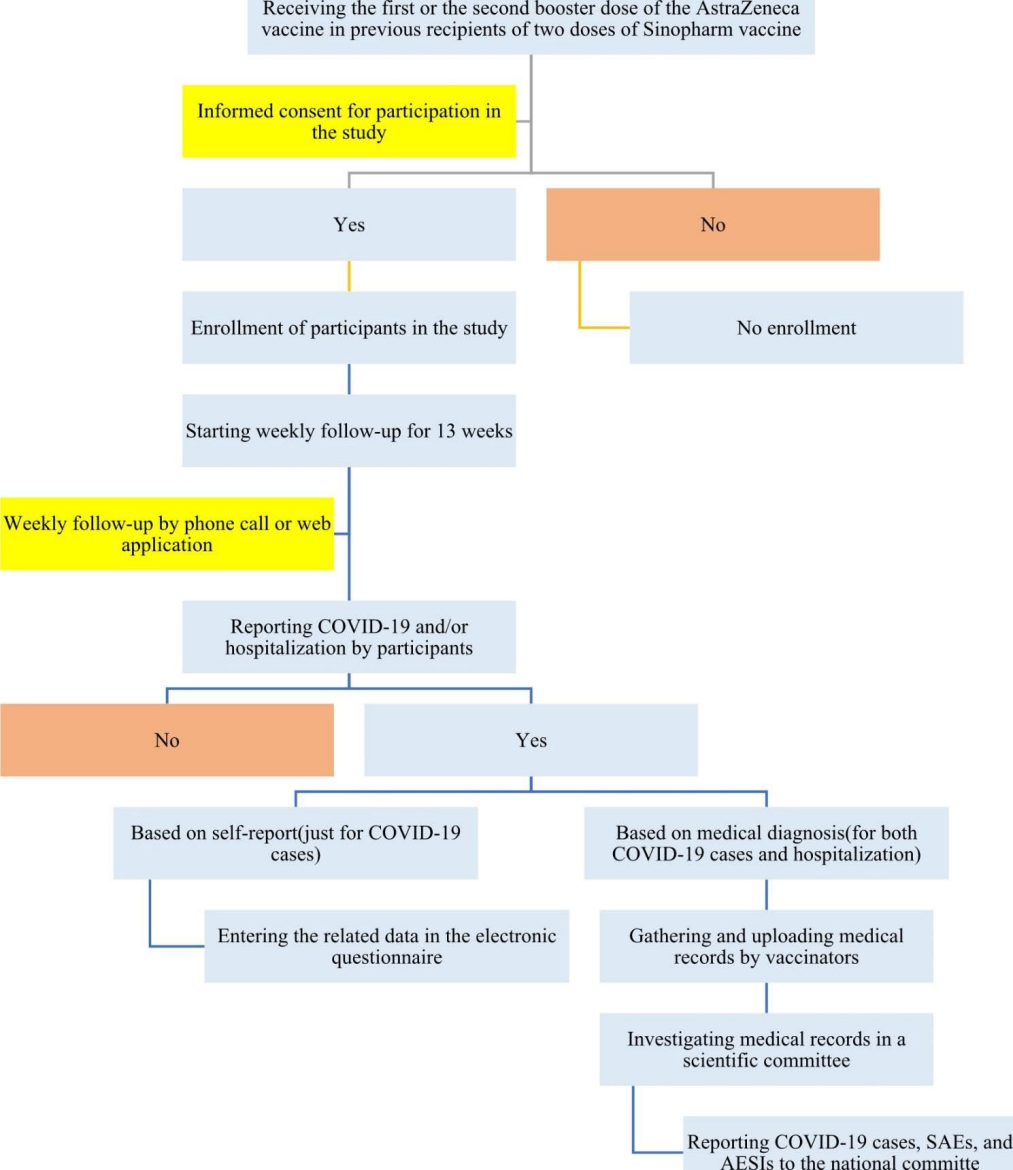
The flow chart of including participants in the present cohort study

The study population includes all enrolled individuals who have received two doses of Sinopharm vaccine and either the first or second booster dose of AstraZeneca according to the national guidelines for immunization against COVID-19 in Iran in 2023. In addition, those who have been vaccinated with two doses of Sputnik V and received the first or second dose of AstraZeneca recruited. Study participation is strictly voluntary, and signed informed consent is obtained from all participants. The process of including participants in the present cohort study is shown in Fig. 1.

#### Inclusion criteria

- Written informed consent.
- Willingness to continue to participate in the study until 3 months.
- Enrolled individuals who have received the primary vaccination with two doses of Sinopharm or Sputnik V and either the first or second booster vaccination with AstraZeneca vaccine.

#### Exclusion criteria

- Individuals unable to comply with study procedures (e.g. People with intellectual disabilities).
- Individuals who have received booster vaccination by AstraZeneca neither as the first nor as the second booster vaccination.
- Those who are less than 18 years old.

### Sample size estimation

Regarding the WHO’s protocol, the sample size required to rule out an event with a frequency of 1 per 3,333 with a 95% confidence interval is 10,000 per vaccine [11]. Therefore, we are planning to include 20,000 and 10,000 eligible people with previous vaccination with Sinopharm and Sputnik V respectively. Regarding the higher proportion of immunization coverage for the Sinopharm vaccine compared to Sputnik V in Iran, we will include more vaccinated people with the Sinopharm vaccine in the present study. Participants will be considered to have completed the study when they have completed the last questionnaire 13 weeks after the booster dose. The end of the study is defined as the point at which the last subject enrolled has reached the 3 months follow-up period. In this study, convenience sampling will be used to include eligible participants until achieving the required sample size.

### Subject recruitment and follow up

People who approach to one of the vaccination centers for booster vaccination, if they are eligible to be included, they will be consulted and get informed about the project. If they accept to participate, they will be asked to sign the consent form. On a day of vaccination, all the required information will be recorded on a web-based questionnaire. For weekly follow up, the study staff will call the participant by phone to collect data if the participant do not use the web application for self-reporting of adverse event. After two unsuccessful attempts to contact the participant by telephone, their next of kin will be contacted. After two unsuccessful attempts to contact their next of kin, the participant will be considered lost to follow-up if they do not complete the questionnaire by self-report. In fact, the participants are able to provide self-reported answers to all web-based questions using the mobile phone or a computer. The attempts to contact will be documented. We will record the underlying reason for withdrawal. Withdrawn participants and those lost to follow-up will not be replaced after the enrolment period has ended.

To identify SAEs and AESIs, each individual will be followed-up for 13 weeks after the booster dose of the AstraZeneca vaccine. The diagnoses reported by the participants during follow-up will be examined by a scientific committee including specialists in the field of infectious diseases, epidemiology, virology/immunology, internal diseases and others if needed. Such committees have been established by each university and they are involving in confirming the diagnosis for all SAEs and AESIs. In addition, there is a national committee to investigate adverse events related to vaccines, and therefore for all SAEs and AESIs, the patients’ files will be discussed in such committee. Such investigation helps participants to be treated within a standard protocol under the supervision of a scientific group.

As per WHO recommendation, all pregnant women inadvertently exposed to COVID-19 vaccine will follow up monthly until delivery, and the pregnancy outcome will be documented if it is possible. Although the available evidence does not fully support the use of AstraZeneca among pregnant women, we do not exclude such participants if they oblige to get a booster vaccination with AstraZeneca. The following maternal AESIs will be investigated among pregnant women: Maternal death and hospitalization, maternal thrombotic events, hypertensive disorders of pregnancy, miscarriage/spontaneous abortion, stillbirth, preterm birth, neonatal death, microcephaly, major congenital anomalies, infant death.

### Study variables

Our independent variables include:

- Age: Calculated as difference between enrolment date and the date of birth for people who will participate in the study.
- Gender (Nominal variable): Male/Female.
- Education (Ordinal variable): Total number of years of education.
- Self-reported underlying health condition (Binary variable: yes/no): An underlying health condition is a chronic or long-term illness, which in turn weakens the immune system.
- Self-reported weight (kg): The body weight for each respondent in our data set measured in kilogram.
- Self-reported height (cm): The body weight for each respondent in our data set measured in centimeter.
- Place of residence: The province where people live.
- Exact date of pervious vaccinations.

Other main variables of the present study gathered in enrollment and follow-up questionnaires is shown in Table 2.

**Table 2:** The main variables of the study gathered in enrolment and follow up questionnaires

Participant information	AESI(Body system)	Follow-up questionnaire
Subject ID	Acute cardiovascular injury(Cardiac)	Hospitalization
Subject Name	Chilblain like lesions(Dermatologic)	Hospitalization date
Phone number	Single organ cutaneous vasculitis(Dermatologic)	Discharge date
Gender	Erythema multiforme(Dermatologic)	Hospitalization reason
Marital status	Pancreatitis(Endocrine)	Hospitalization diagnostics
Birth date	Subacute thyroiditis(Endocrine)	Hospitalization report
Educational attainment	Acute liver injury(Gastrointestinal)	Hospital ID
Income level	Coagulation disorder (thromboembolism)(Hematologic)	COVID-19COVID-19-Test
Place of residence	Thrombocytopenia(Hematologic)	COVID-19 Symptom Onset Date
Pregnancy status	Vaccine-associated enhanced disease (VAED)(Immunologic)	ICU admission
Breastfeeding status	Anosmia, ageusia(Gastrointestinal Respiratory)	ICU admission Date
Name of next kin	Anaphylaxis(Immunologic)	Death
Vaccine dose ID	Acute aseptic arthritis(Musculoskeletal)	Death date
Vaccination date	Rhabdomyolysis(Musculoskeletal)	Reason of death
Vaccine brand	Acute disseminated encephalomyelitis (ADEM)(Neurologic)	Pregnancy
Chronic respiratory disease	Bell’s palsy(Neurologic)	Abortion
Chronic heart disease	Generalized convulsion(Neurologic)	Menstrual irregularities
Chronic liver disease	Guillain-Barré syndrome (GBS)(Neurologic)	
Chronic renal disease	Meningoencephalitis(Neurologic)	
Diabetes	Acute renal injury(Renal)	
Immunocompromised / Immunosuppressed	Acute respiratory distress syndrome(Respiratory)	
Obesity		
Allergy		
Prior Covid		
Date of Prior Covid		
History of reaction to vaccination		
Weight(Kg)		
Height(Cm)		

### Exposure of interest

The exposure of interest is either the first or second booster vaccination with AstraZeneca after the primary vaccination with Sinopharm or Sputnik V. The COVID-19 vaccine brand, dose, vaccination date and batch number will be recorded.

### Study outcomes

#### Serious adverse events (SAEs): hospitalization, death

Serious adverse events (SAEs) is defined as any over-night hospitalization or death. SAEs that lead to in-patient hospitalizations will be reported by the participant or their next of kin, and SAEs that results in death will be reported by their next of kin [12].

#### Adverse events of special interest (AESI) resulting in hospitalization

According to the WHO’s protocol template, the list of AESIs (Table 2) comprises events that have a proven relationship with immunization or a theoretical concern according to immunopathogenesis of COVID-19 disease [12].

#### Sever COVID-19

Severe COVID-19 disease is defined as COVID-19 disease resulting in blood oxygen saturation < 90%, signs of pneumonia and signs of severe respiratory distress. Occurrence of COVID-19 disease will be solicited throughout follow-up to collect information on laboratory-confirmed diagnosis or not [12]. In the present study, the occurrence of COVID-19 is based on clinical manifestations diagnosed by health professionals or/and the polymerase chain reaction (PCR) test.

### Data management

We will develop a data management plan (DMP) before data collection begins and will describe all functions, processes, and specifications for data collection and cleaning. Study staff will enter data in an electronic questionnaire at several time points. The study staff will enter the participants’ information, contact details and data on the exposure at the time of vaccination and all other covariates.

### Data security

In the present study, we stored the key-coded data in a secured database located in Iran. Data will be handled in line with all applicable data protection and privacy laws. No unauthorized persons will have access to the data. Data will be archived for 10 years, as per national regulations, and will then be destroyed. Data will be stored in a secure server environment and will be provided to researchers with an approved proposal by the project data manager. Analyses will be done on de-identified data by authorized researchers.

### Source documents

The data sources for the exposure of interest will be participants and registered data. We should note that by adding the National code for each participant, all required information regarding the previous history of vaccination will be added to the web based questionnaire. The data source for covariates will be the participants as well. The data sources for the study outcomes will be the questionnaires completed by the participants (or their next of kin) as well as documentation from hospital and the universities committees as well as the national committee.

### Data retention and archiving

Documents that individually and collectively permit evaluation of the study conduct and the quality of the data produced will be retained for 10 years in accordance with good pharmacoepidemiological practice guidelines. This will include the analytical data, analysis programs, and all output generated.

### Quality assurance, monitoring, and reporting

For each province, a Co-PI will be selected who has direct communication with the PI and the central team. Remote and on-site monitoring of the study conduct will be performed throughout the study period to assess the accuracy and completeness of the data. The study site may be subject to a quality assurance visit. If so, the site will be contacted in advance to organize a monitoring visit. The investigator and site staff will assure direct access to all study documents for quality assurance monitors. The central team, included the authors of the present study, will control the quality of data once a week to identify errors, outliers and missing data. Furthermore, we will extract 1% of data randomly and contact participants to make sure they are adequately informed about the present study and know how to complete web based follow-up questionnaires. The examination of medical records will be conducted in the KUMS and the national committees. The committees will investigate medical records to approve or reject the association of the booster dose with a health outcome.

### Interim analyses and reporting

For the study outcomes, interim analyses will be performed monthly but the formal report will be released within 1.5, 3, and 6 months. Such reports will be disseminated to all formal organizations and Iranian medical universities (including the Iranian Ministry of Health and Medical Education and WHO by request). In addition, the report will be published in relevant medical journals.

### Final analyses and reporting

Final analyses will be conducted and a full study report will be written within 4 weeks after database lock. Study results will be shared with the national regulatory authorities for regulatory review, and with the national immunization program to inform public health policy decisions.

### Study management

This study will be conducted by the principal investigator, Co-PIs. In addition, scientific advisors contribute to the development of materials, recruitment, training and management of sites, the electronic data capture, and data management and analyses. The Investigator and all study staff will conduct the present study in line with the ethics protocols of the Iran National Committee for Ethics in Biomedical Research. All human resources involved in the conduct of this study will be qualified by education, training, and experience to accomplish their tasks. For the purpose of this study, as well as in person communication with the PI and the central team, the co-PI and all other recruitment staff will take part in two separate training sessions held by the central team and a team from Iranian CDC.

### Statistical analysis

In the present study, the data on SAEs and AESIs will be included for the whole cohort population. People who do not report any event(s) will be considered as participants without event(s). As AESIs can occur even after 42 days following the first booster dose, the sensitivity analysis will be done excluding those AESIs occurring within the first 12 days after the second booster dose.

- We will calculate the frequency and proportion of participants reporting AESIs and SAEs by time since vaccination. For the proportions, 95% confidence intervals will be calculated using an exact method.
- Observed-to-expected analyses will be conducted for SAEs and AESIs with an unknown risk window (most likely situation). The observed incidences for AESIs will be compared with background rates from the most appropriate sources. The expected rate will be age-stratified, and the standardized incidence ratio (SIR) will be calculated.
- Incidence rate of severe COVID-19 disease (any COVID-19 disease diagnosed by a healthcare professional, laboratory-confirmed COVID-19, hospitalization for COVID-19, COVID-19 requiring intensive care unit (ICU) admission, COVID-19 disease resulting in death) will be calculated after either the first or the second booster dose of the AstraZeneca and within the period of follow-up.

The present study will be performed in line with the international ethical guidelines for epidemiology studies published by the Council for International Organizations of Medical Sciences (CIOMS), the Declaration of Helsinki and its amendments, good epidemiological practice (GEP) guidelines and the Iran National Committee for Ethics in Biomedical Research. Data protection and privacy regulations will be strictly observed in capturing, forwarding, processing, and storing individuals’ data. The study protocol and informed consent forms will be reviewed and approved by the Iran National Committee for Ethics in Biomedical Research.

## Discussion

As part of the effort to monitor COVID-19 vaccine safety in Iran, this study aims to evaluate the safety of heterologusbooster vaccination with AstraZeneca in people vaccinated with Sinopharm or Sputnik V vaccines in Iran. The magnitude of the increased risk of serious adverse events after heterologous booster vaccination, if there is any, is still not well understood. Moreover, there are few large cohort studies that identify SAEs among people with heterologous booster vaccination especially among those with vector based or inactivated primary vaccination [13–15]. Accordingly, we decided to set up active COVID-19 vaccine safety surveillance through an observational prospective cohort study in Iran.

To the best of our knowledge, in Iran, there is just an ongoing cohort study by Aliyari et al. for safety signal detection after primary vaccination with COVID-19 vaccines which in turn provides an opportunity to raise awareness of pharmacovigilance among policymakers and healthcare providers, and encourage a perception that pharmacovigilance falls within the scope of a vaccine safety surveillance system [16].

Globally, there are various ongoing studies to investigate the risks of COVID-19 vaccines. For example, Kant et al. (2022), in Netherland, examined differences in the frequencies of ‘well-known’, systemic adverse events following immunization (AEFIs) for four COVID-19 vaccines (AstraZeneca’s Vaxzevria®, Moderna’s Spikevax®, Pfizer’s Comirnaty®, and the Janssen vaccine) in which the frequency of serious AEFIs was the highest for the AstraZeneca (0.228%) and persons receiving the first dose of the AstraZeneca and Janssen vaccines and the second dose of the Moderna vaccine experienced AEFIs mostly [17]. In a study by Consantiono et al.(2022), in Italy, AstraZeneca and Pfizer- BioNTech vaccines indicated a good safety profile but people who received AstraZeneca had a higher number of AEFI than the group who received Pfizer- BioNTech (83% vs. 42%) [18]. In another study (2022) findings supported the safety of the Oxford/AstraZeneca COVID-19 vaccine among healthcare workers in Saudi Arabia so that all the side effects were mild-to-moderate [19]. Also, there are other studies in Africa [20], the USA [6, 21, 22], Canada [23], China, Hong Kong [24, 25], Czech Republic [26], Netherland [27], Italy [28], Korea[29], Spain [30], etc. to explore and measure the causal association between vaccines and adverse events.

The present study can provide a first estimate of the incidence of SEAs and AESI and will give first insights into the incidence of new cases of Covid-19 among the cohort population after heterologous booster vaccination with AstraZeneca vaccine. In addition, it will help to measure the break through infection after booster vaccination.

There are some challenges in the present study. In the last years, the response rates tend to be low in population-based studies considerably [31, 32]. A low response rate might affect the representativeness of the cohort population which might have an effect on the generalizability of the incidence of SEAs and AESI in the Iranian source population.

Regarding that each individual should be followed-up for 13 weeks after either the first or second booster dose of the AstraZeneca vaccine, some participants might be lost to follow up due to a serious outcome such as severe events, hospitalization, or death. Some participants might not tend to be followed up for such a long time of 13 weeks. Also, some individuals may fail to complete the electronic questionnaire weekly because of poor access to a smartphone or forgetfulness. For participants who do not complete the electronic questionnaire regularly, the cohort vaccinators will contact them and fill out the follow-up questionnaire.

One of the major concerns is related to the new coming information regarding the safety of the AstraZeneca and the side effects which might lead to the AstraZeneca refusal among the cohort population [33]. This may in turn decrease our chance to reach the predefined sample size of our study.

Currently, we face remarkable low coverage of third or/and fourth doses of COVID-19 vaccine among the general population in Iran. To date (June 21, 2022), 27,685,680 people have received the third dose of the COVID-19 vaccine, while the amount for the second dose is 57,959,218. Given the notable decreasing trend of the first and second booster doses of vaccination, we might achieve the study sample size (30,000 people) within a longer period of time.

In addition, we will not monitor reactogenicity for either the first or the second booster dose of the AstraZeneca vaccine. In the present study, we just aim to investigate the SAEs (hospitalization, death), AESIs that result in hospitalization, and severe COVID-19 disease.

## Electronic supplementary material

Below is the link to the electronic supplementary material.

Supplementary Material 1

Supplementary Material 2

## Data Availability

The datasets generated and/or analyzed during the current study are not publicly available but are available from the corresponding author on reasonable request.

## Abbreviations

AESI: Adverse events
SAEs: Serious adverse events
SARS-CoV-2: Severe acute respiratory syndrome coronavirus 2
USA: United States of America
WHO: World Health Organization
NIPs: National immunization programs
MOHME: Ministry of Health and Medical Education
CEM: Cohort event monitoring
IPHS: Integrated public health system
DMP: Data management plan
KUMS: Kermanshah University of Medical Sciences
CIOMS: Council for International Organizations of Medical Sciences
GEP: good epidemiological practice
EUL: Emergency Use Listing
WCO: WHO Representative Country Office
