# Supplementary figures and images for: A prospective cohort study protocol: monitoring and surveillance of adverse events following heterologous booster doses of Oxford AstraZeneca COVID-19 vaccine in previous recipients of two doses of Sinopharm or Sputnik V vaccines in Iran
Source: BMC Public Health. 2023 Jul 24;23:1415. doi: 10.1186/s12889-023-16265-8 (PMC10364349; doi:10.1186/s12889-023-16265-8)

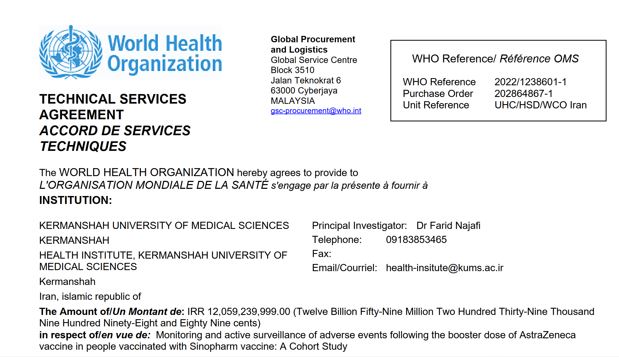

Supplement: Supplementary file 1 — Supplementary Material 1 [file 12889_2023_16265_MOESM1_ESM.jpg]
